# Unravelling the Anticancer Mechanisms of Traditional Herbal Medicines with Metabolomics

**DOI:** 10.3390/molecules26216541

**Published:** 2021-10-29

**Authors:** Omolola R. Oyenihi, Ayodeji B. Oyenihi, Joseph O. Erhabor, Motlalepula G. Matsabisa, Oluwafemi O. Oguntibeju

**Affiliations:** 1 Oxidative Stress Research Centre, Phytomedicine and Phytochemistry Group, Department of Biomedical Sciences, Cape Peninsula University of Technology, P.O. Box 1906, Bellville 7535, South Africa; lolaoyenihi@gmail.com; 2 Functional Foods Research Unit, Faculty of Applied Sciences, Cape Peninsula University of Technology, P.O. Box 1906, Bellville 7535, South Africa; Oyenihia@cput.ac.za; 3 Department of Pharmacology, University of the Free State, P.O. Box 339, Bloemfontein 9300, South Africa; joseph.erhabor@uniben.edu; 4 Phytomedicine Unit, Department of Plant Biology and Biotechnology, University of Benin, PMB 1154, Benin City 300001, Nigeria

**Keywords:** cancer, herb, metabolic reprogramming, metabolomics, phytomedicine

## Abstract

Metabolite profiling of cancer cells presents many opportunities for anticancer drug discovery. The Chinese, Indian, and African flora, in particular, offers a diverse source of anticancer therapeutics as documented in traditional folklores. In-depth scientific information relating to mechanisms of action, quality control, and safety profile will promote their extensive usage in cancer therapy. Metabolomics may be a more holistic strategy to gain valuable insights into the anticancer mechanisms of action of plants but this has remained largely unexplored. This review, therefore, presents the available metabolomics studies on the anticancer effects of herbal medicines commonly used in Africa and Asia. In addition, we present some scientifically understudied ‘candidate plants’ for cancer metabolomics studies and highlight the relevance of metabolomics in addressing other challenges facing the drug development of anticancer herbs. Finally, we discussed the challenges of using metabolomics to uncover the underlying mechanisms of potential anticancer herbs and the progress made in this regard.

## 1. Introduction

The heterogeneous nature of tumour cells and the genetic diversity in cancer patients have compelled the re-assessment of the magic bullet approach in combating cancer. There is an increasing interest in multi-targeted therapies to enhance the pharmacological efficacy of anticancer drugs and limit chemo-resistance. This involves simultaneous actions of multiple chemicals on many molecular targets or synergistic actions on a single site. Medicinal plants are being explored as valuable sources for drug discovery due to their production of a cocktail of phytochemicals that often act in concert to afford some biological properties. About 80% of anticancer drugs approved by the United States Food and Drug Administration (US FDA) in the last decades were natural products or derivatives [1]. However, there is a declining interest in their development in the pharmaceutical industry as the reliance on chemically synthesized compounds for cancer drug discovery grows [2]. One major challenge in developing new plant-derived pharmaceuticals is unclear or unknown biochemical and pharmacological mechanisms of action. The difficulty in evaluating the precise mechanism of action coupled with other challenges such as authentication of plant material, toxicity concerns, and challenges in the large-scale production of lead phytochemicals due to insufficient plant material have slowed progress in the drug discovery from plants [3].

Understanding the mechanism of action of medicinal plants requires elucidation of the interactions between complex mixtures of phytoconstituents and possible targets (cellular and molecular) that generate the pharmacological response. While target based-screens offer numerous advantages for drug discovery, it involves a trial-and-error approach, which allows a partial understanding of the mechanisms of action. In some cases, target inhibition may not translate into the desired pharmacological effect or efficacy possibly due to a different target from that initially expected [4]. As a result, only a small percentage of drugs make it past phase 1 trials, while most fail due to lack of efficacy, off-target activity, and toxicity issues. Most clinically approved plant-derived anticancer compounds received approval decades after the initial identification of their medicinal effect (Table 1). Sadly, this timeframe has not changed significantly in recent times.

Despite the enormous research on anticancer medicinal plants in the last decades, the number of new plant-derived drugs has not increased proportionately. However, herbal medicine practices continue to be an integral aspect of the local culture of healthcare delivery. About 80% of Asian and African populations rely on traditional medicine for their health care needs. The past decade has witnessed a surge in the acceptance of herbal medicine as a reliable source of healthcare due to its affordability, accessibility, perceived safety, and efficacy [5]. The observations that some herbal preparations show superior effects to single chemical constituents at the equivalent concentration have raised the scientific interest in studying the pharmacological effects of herbal exposure. In both developed and developing countries, herbal preparations and products are used alone or combined with conventional anticancer drugs to improve their anticancer effects [6]. Therefore, research into traditional herbal medicines’ anticancer mechanisms and toxicity profiles should be encouraged to provide in-depth scientific underpinnings for their use and provide information on potential herb-drug interaction to ensure patient safety.

Metabolomics, a term coined by Steven Oliver in 1998, allows for simultaneous multi-metabolite analysis in biological samples [7]. The concept of metabolome analysis fits the holistic concept of traditional herbal medicine and could provide further insight or scientific underpinnings into the anticancer mechanism of action of herbal medicines. Here, we summarize metabolic changes in cancer cells and present an overview of the metabolomics studies on the cytotoxic effects of traditional Chinese, Indian, and African herbal medicine systems. We also present some scientifically understudied ‘candidate plants’ for cancer metabolomics studies. We highlight the relevance of metabolomics in addressing other challenges facing the development of anticancer agents from herbs. Finally, we present the challenges and progress in applying metabolomics to elucidate the underlying mechanisms of anticancer herbs.

**Table 1:** FDA-approved plant-derived anticancer drugs; time frame of approval and mechanisms of action.

FDA Approved Drug/Year	Initial Discovery	Plant Source	Cancer Type	Mechanism of Action	References
Paclitaxel/1992	1960s	*Taxus brevifolia* Nutt. (bark)	Breast, ovarian, lung, pancreatic	Stabilizes microtubule	[8]
Homoharringtonine/2012	1970s	*Cephalotaxus harringtonii* (Knight ex J.Forbes) K.Koch (bark)	Chronic myeloid leukaemia	Disables the elongation of peptide chain inhibiting protein synthesis	[9]
Camptothecin/1996	1960s	*Camptotheca acuminata* Decne (bark and stem)	Gastrointestinal, ovarian, small-cell lung	Inhibits deoxyribonucleic acid (DNA) re-ligation through interaction with topoisomerase-type I DNA complex causing DNA damage	[10]
Vincristine sulphate/1963	1950s	*Catharanthus roseus* (Linnaeus) G.Don (leaf)	Leukemia	Inhibits the formation of microtubules and interferes with nucleic acid and protein synthesis by blocking glutamic acid utilization	[11]
Vinblastine sulphate/1965	1950s	*Catharanthus roseus* (leaf)	Lymphoma, choriocarcinoma, breast	Inhibits microtubule formation resulting in cell cycle arrest	[11]
Teniposide (semisynthetic analogues of podophyllotoxin)/1990	1960s	*Podophyllum peltatum* Linnaeus (rhizome)	Leukaemia	Inhibits type II DNA topoisomerase complex	[12]
Etoposide (semisynthetic analogues of podophyllotoxin)/1983	1960s	*Podophyllum peltatum* Linnaeus (rhizome)	Testes, lung	Inhibits type II DNA topoisomerase complex	[13]

## 2. Metabolomics: An Indispensable Tool for Anticancer Drug Discovery from Plants

**Figure 1:**
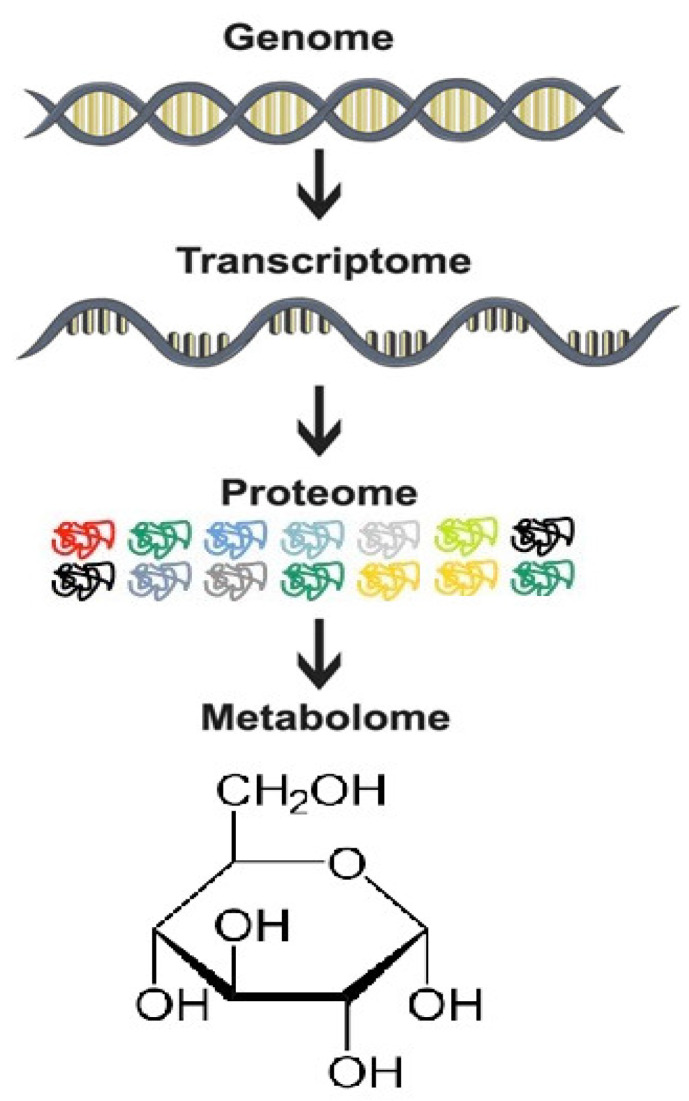
The omics cascade. The metabolome represents the final step of the omics cascade and is downstream of the proteome. Any alteration in the metabolome triggered by drug treatment would therefore provide information on the effects of the drug.

Altered metabolism is one of the hallmarks of cancer, where changes in gene expression and protein functions resulting from genetic and epigenetic alterations ultimately lead to aberrant cellular metabolism. The metabolome is a complement of metabolites (such as glucose, lactate, pyruvate, amino acid, or lipid signalling molecules, etc.) involved in intermediary metabolism and whose levels are related to genetic expression (Figure 1). Metabolome reflects changes in enzyme activities, alteration in signalling pathways, catabolic and synthetic reactions [14].

Metabolomics may offer a more productive route to anticancer drug discovery from plants due to several reasons:

- Genomics, epigenetics, proteomics, and transcriptomics ultimately converge to metabolomics, making metabolic profiling indispensable to uncover the molecular target of medicinal plants [15].
- Analyses of subtle changes in metabolite levels in many metabolic pathways aid in hypothesis generation, enabling a top-down approach in discovering mechanisms of drug action [16].
- In the investigation for anticancer efficacy of potential therapeutics, metabolite biomarkers are stable end products that may be more reliable as indices of the initiation or progression of cancers than mRNA or proteins [17].
- Metabolomics offers the possibility of conducting non-invasive, large-scale studies (using bio-fluids like plasma, serum, and urine samples) to determine the efficacy of medicinal plants and their chemopreventive potential in the malignant transformation of normal cells [18].
- Metabolomics offers the opportunity to decipher the pharmacokinetics of herbal medicines and possible interactions with conventional anticancer drugs [19]. It is a tool to assess potential drug toxicity and to avoid drug withdrawals in early-phase clinical trials due to toxicity concerns [20]. These pharmacological evaluations are essential during the long-term use of traditional herbal medicines.
- Metabolic profiling is useful for the authentication and standardization of traditional herbal medicines [21]. It can be a quick preliminary guideline to uncover the most dominant compound related to the anticancer activity or predict potential herb-drug interactions and toxicity. Besides, the seasonal variations in plant components can also be studied using metabolomics [22].

The two main approaches in metabolomics are targeted (biased) and untargeted (unbiased) metabolite profiling. The targeted approach is aimed at a pre-defined panel of metabolites in a biological sample, whereas the untargeted approach examines the complete metabolome providing a holistic assessment of metabolite composition in samples [23]. Both approaches employ analytical techniques such as nuclear magnetic resonance (NMR) spectroscopy and mass spectrometry (MS). A considerable number of detailed procedures abounds elsewhere [24,25] with corresponding technical reviews [26,27] on the application of these metabolomics tools for further exploration.

## 3. Cancer Metabolic Reprogramming

### 3.1. Addiction to Glucose, Glutamine, and Other Amino Acids

To evaluate the alterations in metabolism that are induced by cancer cells, Otto Warburg observed that these cells constitutively metabolize glucose via glycolysis and produce lactate even in the presence of abundant oxygen [28]. This concept of aerobic glycolysis is known as “Warburg’s effect”. Normal cells respond differently by activating glycolysis in response to hypoxia but undergo respiration (oxidative phosphorylation) during oxygen availability. Warburg proposed that this metabolic shift to aerobic glycolysis in cancer cells is due to a mitochondrial respiratory defect [29]. However, it has been observed that many cancer cells also actively maintain mitochondria respiration to generate tricarboxylic acid (TCA) cycle intermediates for macromolecule synthesis [30]. Enhanced glycolysis in cancer cells under hypoxic conditions is necessary to supply glycolytic intermediates and synthesis of macromolecules to meet the demand of the highly proliferating cells [31]. The expression of glucose transporter 1 (GLUT1) is under the positive regulation of hypoxia-inducible factor (HIF)-1 [32]. The hypoxic tumor environment induces GLUT1 expression in a HIF-1-dependent manner that leads to an increase in cellular glucose uptake and promotes the aerobic glycolysis of cancer cells, which enhances the proliferation and metastasis of cancer cells.

Elevated dependence on glutamine is another metabolic characteristic of many cancer cells. Glutamine is a non-essential amino acid derived extracellularly or synthesized endogenously by glutamine synthetase when an exogenous supply of glutamine is scarce [33]. Cancer cells are highly dependent on the exogenous supply of glutamine mediated by several solute carrier groups of transporters. Following its entry into the cell, glutamine has different metabolic actions. Glutamine is catabolized to glutamate by the mitochondrial enzyme glutaminase (GLS). The amino and amide nitrogen groups from glutamine are crucial to producing other non-essential amino acids like serine and glycine. The carbon skeleton from glutamine catabolism contributes to the carbon pool to replenish the TCA cycle (anaplerosis), supporting the production of adenosine triphosphate (ATP) and the biosynthesis of protein, nucleotides, and lipids [34]. Glutamine-derived glutamate and cysteine are required for de novo synthesis of the antioxidant tripeptide-glutathione (GSH). GSH combat oxidative stress, thus protecting cells from the damage caused by excessive generation of reactive oxygen or reactive nitrogen species [35]. Increased rate of glutaminolysis contributes to the biosynthesis of NADPH, a reducing agent needed to fulfil the requirements for cell proliferation and regenerate GSH from its oxidized form (GSSG) [36].

### 3.2. Enhanced Lipid Utilization

As components of biological membranes, lipids play a role in maintaining membrane structure and are also important signalling molecules. The alterations in fatty acid transport-enhanced de novo lipogenesis and β-oxidation are metabolic characteristics of cancer cells [37]. Cancer cells obtain lipids through direct exogenous uptake from the surrounding microenvironment via specialized transporters such as fatty acid translocase and fatty acid transport protein family whose expressions are increased in the diseased state. Exogenous uptake of fatty acids allows for metabolic flexibility within cancer cells. Lipid uptake may be essential during conditions of metabolic stress when the ability to meet oncogene-driven demands for biosynthesis is compromised [38]. Cancer cells also obtain lipids through de novo synthesis to maximize lipogenesis or protect cancer cells from oxidative lipid damage [39].

Overexpression of fatty acid biosynthetic genes such as those encoding for fatty acid synthase (FASN), ATP-citrate lyase (ACLY), and acetyl-CoA carboxylase (ACC), have been reported in tumors of various cancer types [40]. Fatty acid synthesis occurs via integration with other carbon metabolism pathways such as glycolysis or glutaminolysis to obtain acetyl-CoA and NADPH. Also, fatty acids can supply substrates to the TCA cycle to sustain mitochondrial ATP production in cancer cells. The β-oxidation of fatty acids in the mitochondria generates acetyl-CoA and the reducing equivalents NADH and FADH2, which are critical for generating mitochondrial ATP by the electron transport chain [41]. The multiple inputs of metabolites from glycolysis, glutaminolysis and β-oxidation of fatty acids into the TCA cycle equip cancer cells with metabolic flexibility to adapt to nutrient availability. This metabolic rewiring is not only needed to fuel energy needs or to support biomass generation but plays essential roles in other cancer features such as migration, invasion, and metastasis.

### 3.3. Oncogenic Activation of Metabolic Pathways

Metabolic reprogramming results from mutations in oncogenes and tumor suppressors, and the activation of signaling pathways that directly contribute to malignant transformation. Alterations in the gene expression of key enzymes of metabolic pathways support oncogenic transformation and tumor progression. Oncometabolites, detected in elevated levels in tumors, sustain tumor growth and metastasis via modulation of cell signaling and epigenetic modifications. Several oncogenes, including MYC, phosphoinositide-3-kinase (PI3K), KRAS, protein kinase B (AKT), mechanistic target of rapamycin (MTOR), and HIF-1, are implicated in the regulation of cancer metabolic reprogramming [42].

The P13K/AKT signaling pathway is a commonly dysregulated pathway in cancer. Over-stimulated P13K/AKT enhances glycolysis, mitochondrial biogenesis, and activates other anabolic pathways involved in the de novo biosynthesis of fatty acids, nucleotides, and proteins, ultimately promoting cell survival [43]. Over-activation of this pathway occurs independently of the extrinsic growth factor through various molecular mechanisms, including mutations of receptor tyrosine kinases (such as EGFR, HER2), tumor suppressor genes, or components of the PI3K complex [44]. The activated phosphotyrosine residues of the receptor tyrosine kinases (RTK) interact with src-homology 2 domains on PI3K, leading to the generation of the lipid second messenger, phosphatidylinositol 3,4,5-trisphosphate (PIP3) from phosphatidylinositol-4,5-bisphosphate (PIP2). AKT localizes to the cell membrane through interactions with PIP3, which ultimately leads to phosphorylation and activation of AKT [45]. Downstream of PI3K/AKT is the cell growth regulator, the mTOR complex 1 (mTORC1). Mutations in the MTOR gene itself can directly lead to its activation. The mTORC1 protein is also more commonly activated downstream of gain-of-function mutations in the PI3K/AKT pathway or through inactivation of tumor suppressors such as phosphatase and tensin homolog (PTEN). It coordinates the expression of other oncogenes and transcription factors, which play key roles in controlling intracellular metabolism. Specifically, the mTOR pathway stimulates glutaminolysis by upregulating the expression of CMYC, which induces the expression of glutamine transporter and GLS [46]. The gain of function mutation by MYC also enhances glycolysis through the amplified expression of genes that support tumor proliferation, including transporters and enzymes involved in glycolysis, glucose uptake, lactate production and export, fatty acid synthesis, serine metabolism, and mitochondrial metabolism [47]. Additionally, the oncogene-directed activation of glycolysis also occurs through HIF-1α, which coordinates the metabolic adaptation to hypoxia [48]. The activation of mTOR is also known to play a role in the stabilization of HIF-1α protein, which can lead to enhanced activation of their transcriptional targets, including GLUT1, and other glycolysis enzymes. The PI3K/AKT oncogenic signalling pathway also activates SREBP-1, a transcriptional factor that controls lipogenesis. SREBP-1-mediated de novo lipogenesis is a critical component of mTORC1-driven proliferation [49]. Activated AKT can also directly stabilize the nuclear SREBP-1, thus promoting its target gene expression.

**Figure 2:**
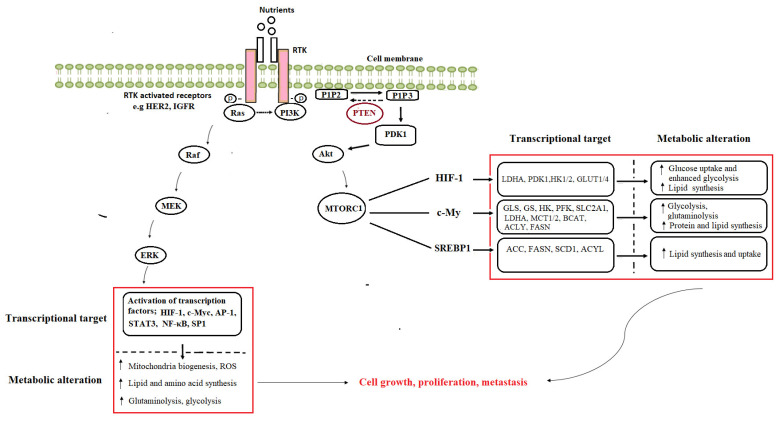
The oncogene-induced activation of metabolic pathways in cancer. GLS—glutaminase, GS—glutamine synthetase, HK—hexokinase, LDHA—lactate dehydrogenase A, MCT—monocarboxylate transporter, PDHK—pyruvate dehydrogenase kinase, SLC—solute carrier family, PFK—phosphofructokinase, GLUT—glucose transporter, ACC—acetyl-CoA carboxylase, ACLY—ATP citrate lyase, BCAT—branched-chain aminotransferase, FASN—fatty acid synthase, SCD—stearoyl-CoA desaturase, HIF—hypoxia-inducible factor, SREBP—sterol-response element-binding protein, mTORC—mammalian target of rapamycin complex, AP—activator protein, STAT—signal transducer and activator of transcription, NF-κB—nuclear factor kappa B, SP—specificity protein, ROS—reactive oxygen species, Akt—protein kinase B, PDK—phosphoinositide-dependent kinase, PIP2—phosphatidylinositol-3,4-biphosphate, PIP3—phosphatidylinositol-3,4,5-trisphosphate, PTEN—phosphatase and tensin homolog deleted on chromosome 10, RTKs—receptor tyrosine kinases, PI3K—phosphoinositide-3-kinase, ERK—extracellular-signal-regulated kinase, MEK—MAPK/ERK kinase, RAF—rapidly accelerated fibrosarcoma.Many pharmacological agents targeting cancer metabolic vulnerabilities are at different stages of drug development, as shown in Table 2.

Aside from oncogenes, tumor suppressors such as the p53 and PTEN, which regulate metabolism, are frequently mutated or deleted in many human cancers. PTEN executes its tumor suppressor activity by dephosphorylating PIP3 to PIP2, thereby counteracting PI3K signaling and inhibiting AKT-dependent pathways. The inhibition of PI3K/AKT signaling suppresses glycolysis, several anabolic pathways, and mitochondrial metabolism [50]. Also, the tumor-suppressive functions of p53 are related to the execution of DNA repair, cell cycle arrest, apoptosis induction, regulation of metabolism, and oxidative stress [51]. Loss of p53 and PTEN promotes tumorigenesis by increasing glycolytic flux and redox imbalance. Other signaling pathways e.g., the RAS/RAF/MEK/ERK cascade downstream of oncogenes and tumor-suppressor genes can also regulate cancer metabolism. The ERK pathway is activated by a series of phosphorylation events that occur downstream of various activated receptor types, including RTKs in response to extracellular stimuli such as growth factors [52]. Constitutive activation of ERK signaling in many cancers is caused by factors such as overexpression or mutation in receptor tyrosine kinases, Ras and Raf mutations, sustained autocrine or paracrine production of activating ligands, and the direct amplification or deregulation of its transcription factor targets, such as MYC, HIF-α, activator protein 1 and signal transducers and activators of transcription [53]. These targets are involved in the transcriptional regulators of glycolysis, glutaminolysis and nucleotide and fatty acid synthesis [52], as summarized in Figure 2.

## 4. Medicinal Plants as Modulators of Cancer Metabolism

Medicinal plants consist of many bioactive compounds that can target multiple dysregulated, but cross-linked pathways in cancer. These include pathways involved in the metabolism of carbohydrates, amino acids, and lipids. Many plant-derived compounds and extracts inhibit metabolic enzymes and transporters, PI3K/AKT oncogenic signaling, and the Ras/Raf/MEK/ERK/MAPK pathways, thereby restricting cancer progression. In many cancers, the overexpression of metabolic targets (e.g., enzymes and transporters) offers a relatively safe therapeutic window for anticancer agents [66]. Table 3 shows a list of plant extracts/compounds and their metabolic targets in cancer cells and tumors and illustrates the spectrum of potential anticancer herbal intervention points, which encompasses metabolic enzymes, molecular transporters, oncogenes, tumour suppressors, and transcription factors. Cancer metabolome analysis could offer a comprehensive understanding of metabolite changes and novel insights into medicinal plants’ anticancer effects, which would direct further mechanistic studies.

## 5. Deciphering the Anticancer Mechanisms of Traditional (African Chinese, Indian) Herbal Medicines with Metabolomics

Traditional Chinese Medicine (TCM), Traditional Indian Medicine (TIM) (represented by Ayurveda), and Traditional African Medicine (TAM) are the most widely used traditional medicine systems today [95]. These ancient healthcare systems embrace herbal remedies prescribed as single herbs or mixtures of many herbs, as a cornerstone of traditional medicine. Polyherbal formulations usually consist of the primary herbs (responsible for the main therapeutic effect) and secondary herbs (which enhances the action of the primary herbs, reduce side-effects, or improve the palatability of the herbal formulation).

A continuous effort by China in promoting its indigenous therapies using a science-based approach has put TCM in a commendable position and increased the number of approved Chinese medicine for the treatment of various ailments. Chinese herbs are used increasingly as an alternative to conventional cancer treatments while Indian Ayurvedic medicine is also gaining popularity globally [96,97]. The TAM is a diverse and multifaceted knowledge system transmitted through generations in oral form. Some African medicinal plants have found a global market in Europe and the United States e.g., Devil’s claw (*Harpagophytum procumbens*) and *Pelargonium sidoides* DC for treating rheumatic and upper respiratory conditions [95,98].

Cell, animal, and clinical studies have demonstrated the anticancer effects of many herbal constituents of AHM, IHM, and CHM. However, these herbs’ molecular mechanisms of action and formulations are still largely unknown due to the complex chemical components and multiple molecular targets. A holistic approach is therefore needed for a comprehensive evaluation of the efficacy, mechanisms of action and safety in well-controlled experimental models and clinical trials. Metabolomics is applied to decipher the multitarget action of some of these herbal medicines on cancer metabolome as discussed below.

### 5.1. Curcuma Longa Linnaeus

Studies have shown that an ethanol extract of the rhizomes of *Curcuma longa* (Family; Zingiberaceae, common name: turmeric) and polyherbal formulations containing *Curcuma longa* and other ayurvedic herbs reduced tumour size while offering a remarkable symptomatic relief in cancer patients with no adverse effects [99,100]. The cancer-preventive and therapeutic effects were also examined in different experimental models [101].

To gain a deeper mechanistic insight into the action of *Curcuma longa*, Zhou and colleagues recently studied the integration effects of curcuminoids (major compounds in *Curcuma longa*) on A549 lung cancer cells [102]. The metabolic effect of curcuminoids mixture and three individual curcuminoids with natural proportion in turmeric was investigated on A549 lung cancer cells. Although overlap in the metabolic alterations was noted, synergistic and antagonistic actions between the curcuminoids resulted in a shift towards a more positive profile in phospholipids and sphingolipids metabolism than in the individual curcuminoid-treated cancer cells. The data suggest that the use of multi-curcuminoids rather than each compound is more effective on the metabolic alterations of A549 cells. This is in line with a report of superior anticancer activity of total extract of *Curcuma longa* compared to its curcuminoids on A549 human lung cancer, HT29 colon cancer, and T98G glioblastoma cell lines [103].

An earlier study on the toxicity of curcumin on MCF7 and MDA-MB-231 breast cancer cells identified prominent targets of curcumin as lipid and glutathione (GSH) metabolism [104]. Relative DNA content decreased while DNA damage increased with dose and duration of exposure to curcumin. At a low dose, curcumin increased GSH levels but reduced GSH levels and related metabolites at a high dose. The authors further established the significance of their findings in metabolic fuel homeostasis by measuring glutathione-S-transferase (GST) activity in cells exposed to increasing concentrations of CUR. The changes in GSH and GST indicate the modulatory effects of curcumin on the cellular redox status.

Furthermore, at high doses, changes in lipid profile—a decrease in glycerophospho-ethanolamine (GPE) and -choline (GPC), and an increase in polyunsaturated and total free fatty acids were recorded [104]. The accumulation of free fatty acids may be due to deficient cellular mitochondrial functions and the inability of the cells to oxidize fatty acids. The decrease in GPE and GPC by-products of phospholipase A2 (PLA2) enzyme suggest downregulation of PLA2 activity possibly as a means for the cell to limit the release of membrane fatty acids and propagation of oxidative stress. Data from the metabolomics analysis also provides information on the toxic mechanisms of curcumin on cancer cells. Similar metabolic effects observed in both MCF-7 and MDA-MB-231 breast cancer cell lines despite differences in the expression of hormonal receptors suggest that the metabolic targets of curcumin are not dependent on hormonal signalling pathways. The biphasic or hormetic modifications in glutathione metabolism, lipid metabolism, and glucose utilization in cells treated with curcumin alone or in combination with docetaxel, depending on the dose and treatment duration, may explain the paradoxical effects reported for CUR at different doses in various therapeutic combinations and cell types.

### 5.2. Zingiber Officinale Roscoe

*Zingiber officinale* is a component of several Indian and Chinese herbal formulations used for cancer treatment. Examples of such herbal formulations are Immunotone, Cancertame, Xiaoyao powder, and Renshen Yangrong decoction [105,106]. In combination with honey, the ground root is used to treat different cancers in North Africa [107]. Clinical trials and in vitro and animal experiments support the use of ginger as an anticancer herb. Ethanolic extract of its rhizome (1.65–250 μg/mL) induced cytotoxicity of the cholangiocarcinoma cell line, CL-6, [108]. Aqueous suspension of the rhizome powder (50 mg/kg b wt/day for 15 weeks) reduced the colon cancer risk in rats via its hypolipidemic and antioxidative effects [109]. In a clinical study, pure encapsulated ginger powder supplementation (2 g/day for 28 days) reduces the proliferation of colorectal epithelium in patients at increased risk for colorectal cancer [110].

Parvizzade and colleagues studied the anticancer effect of methanolic ginger extract on the metabolic phenotype of Raji cells (lymphoblastoid cells derived from a human Burkitt (non-Hodgkin) lymphoma) [111]. The results showed that the ginger extract displayed significant cytotoxicity on Raji cells and affected both amino acid and carbohydrate metabolism. The significance of these metabolic signatures is worth further evaluation.

### 5.3. Glycyrrhiza Species (G. glabra L, G. uralensis Fisch. and G. inflata Batalin)

Glycyrrhiza (Family: Leguminosae), commonly known as licorice, is a plant with a long history as a food flavouring agent. The most studied among over 30 known *Glycyrrhiza* species are *G. uralensis*, *G. inflata*, and *G. glabra*. According to the 2015 edition of the Chinese Pharmacopoeia, the roots of these species are all identified as licorice without discrimination, despite species variation in chemical constituents [112]. *G. glabra* is used as monotherapy by cancer patients in Northern Africa for blood and lung cancers [113]. *G. uralensis* is a component of PHY906, a polyherbal Chinese formulation currently being developed as an adjuvant for chemotherapy [114]. Several studies (preclinical and clinical) have indicated that PHY906 enhances the antitumor efficacies of a broad spectrum of anticancer agents but showed cytoprotective effects in non-cancer cells [115]. Data from many experimental studies suggest that *G. uralensis* and *G. glabra* may be potential anticancer herbs [116,117].

Cancer cell metabolomics analysis has only been carried out on *G. glabra* (Aqueous root extract)-treated C666-1 nasopharyngeal carcinoma cells. Following treatment, the cells displayed altered amino acid metabolism, evident by a decrease in glutamine, L-alanine, glycine, and L-serine levels, which the authors suggest may be associated with the anti-tumour activity. Elevated levels of these amino acids are found in the sera of nasopharyngeal carcinoma patients [118]. L-alanine acts as an alternative carbon source by outcompeting glucose and glutamine-derived carbon. Glycine and serine supply precursors for biosynthetic pathways of proteins, nucleic acids, and lipids. The hyper-activation of glycine and serine synthesis drive oncogenesis [119,120].

Furthermore, *G. glabra* root extract decreased the levels of glutathione—a tripeptide that plays a role in protecting cells from damage caused by reactive oxygen or reactive nitrogen species [35]. ROS is a “two-edged sword” in cancer. While it supports tumor progression, its production must be regulated at a certain threshold to avoid cancer cell killing due to excessive oxidative stress [121]. In this light, the accumulation of ROS by *G. glabra* via interference with the cellular ROS scavenging system could explain some of its anti-cancer effects. It may be worth evaluating the possible species-specific effects of *G. glabra* and *G. uralensis* on cancer metabolome in future studies.

### 5.4. Nigella sativa Linnaeus

*Nigella sativa* (Family: Ranunculaceae), commonly known as black cumin, is the most prescribed plant for cancer treatment in North Africa. The seeds are traditionally used to treat several ailments including, but not limited to cancer [122]. It is prescribed as monotherapy or ingested with honey for different cancer [107,113]. The seed extracts have displayed cytotoxicity against breast, lung, and colon cancer cells [123,124]. Also, the essential oil reduced solid tumor volume and cancer metastasis and improved survival rate in mice [125] while reducing the side effects of conventional therapy in patients with acute lymphoblastic leukemia [126].

Thymoquinone, a primary component of the essential oil from the seeds, altered the phospholipids metabolism in Jurkat cells and caused an accumulation of ceramide [127]. Ceramide is a bio-effector molecule that mediates cancer cell death, senescence, and cell cycle arrest [128]. Ceramide accumulation in cancer cells is associated with the downregulation of AKT signaling leading to apoptotic cell death [129]. Many tumors exhibit increased ceramide metabolism mainly by increasing activities of sphingolipid enzymes, such as glucosylceramide synthase, ceramide kinase, acid ceramidase, and sphingosine kinase, which result in increased synthesis of sphingolipids with pro-survival effects [128]. A decrease in glutamine and α-ketoglutarate was also observed in thymoquinone-treated HL-60 leukemia cells regardless of either of the two doses tested (5 µM and 10 µM) but not in Jurkat cells where the opposite effect was noted at the 10 µM dose. This data highlights the importance of formulating cancer-specific herbal medicines. It will also be interesting to evaluate the effects of *Nigella sativa* extracts on metabolic signatures of cancer cells in future studies.

### 5.5. Crithmum maritimum Linnaeus

*Crithmum maritimum* belongs to the Apiaceae family of plants and is well known as rock samphire or sea fennel. It has both nutritional and medicinal value in many parts of the world. Although its antitumorigenic effects are well reported [130], there is a scarcity of information on its mechanism of action. Ginochi et al. [131] expanded on their previous study showing a significant growth inhibitory effect of *Crithmum maritimum* on HCC cells via cell cycle regulation and apoptosis. Using a metabolomic approach, the authors showed that the ethyl acetate extract counteracts the Warburg effect in two HCC cell lines (Huh7 and HepG2) by reducing intracellular lactate. A substantial decrease in several amino acids in treated cells suggests the inhibitory effect of *Crithmum maritimum* on protein anabolism. These data demonstrate that *Crithmum maritimum*-induced cytostasis is exerted through a multi-effect action, targeting metabolic processes in HCC cells.

### 5.6. Cyperus rotundus Linnaeus

*Cyperus rotundus* (Cyperaceae family) is commonly referred to as Xiangfu or Nagarmotha in Chinese and Ayurveda medicines respectively, where it is used for breast cancer treatment. The rhizomes are ground into powder and mixed with ginger juice [132]. *Cyperus rotundus* is also a component of some polyherbal formulations available in the market for cancer treatment, such as Chaihu-Shugan-San and US6780441B2 [105,133]. Ethanol extract from the dry rhizomes of *Cyperus rotundus* caused cell cycle inhibition and induced apoptosis in MDA-MB-231 triple-negative breast cancer cells [132].

With the aid of metabolomics, a study recently reported that *Cyperus rotundus* likely induced apoptosis in TNBC cells by causing an arrest of aerobic glycolysis and increasing the pathways of ATP-consumption like amino acids metabolism, fatty acid metabolism, riboflavin metabolism, and purine metabolism, consequently leading to ATP depletion and cell death [134]. These altered metabolic profiles can serve as a basis for further hypothesis formulation in mechanistic follow-up studies to assess its effects on some key players (enzymes, protein) in these metabolic pathways.

## 6. Understudied Herbal Medicines as Potential Candidates for Cancer Metabolomics Studies

The existing data to date indicates that metabolomics-based studies on the anticancer effects of medicinal plants are limited. Many scientifically understudied CHM, IHM and AHM are candidates for metabolomics studies. Several anticancer botanicals and formulations are yet to be scientifically validated or remain largely understudied, leaving more opportunities for drug discovery. Of particular interest are highly sought-after herbal medicines that have significant cytotoxic activities on cancer cells but display low, if any, observable physiological toxicities [135,136]. Examples of such plants are *Sutherlandia frutescens* (Linnaeus) R.Br., *Albizia adianthifolia* (Schum.) W.Wight, *Hypoxis hemerocallidea* Fisch., C.A.Mey. &Avé-Lall., *Coix lacryma-jobi* Linnaeus., *Terminalia arjuna* (Roxb. ex DC.) Wight & Arn, *Artemisia annua* Linnaeus and *Prunus africana* (Hook.f.) Kalkman. The traditional uses of these highly sought-after plants for cancer treatment are well documented [137,138,139,140].

For example, *Sutherlandia frutescens* (commonly referred to as cancer bush) is an indigenous South African medicinal plant highly commercialized as an immune booster. Infusions from the leaves and stems of these plants are traditionally used for cancer treatment [137]. A few scientific reports also exist to support its anticancer effects [141]. Interestingly, several studies have reported that its usage is not associated with any physiological toxicity, strengthening the rationale to evaluate its anticancer efficacy and mechanism of action further [135].

Kanglaite injection is an oily extract from the seed of *Coix lacryma-jobi*, and the first TCM that was approved for a phase III clinical trial by the US FDA in 2015 [142,143]. Studies have shown its potential to reverse multiple-drug resistance and enhance the sensitivity of tumor cells to chemotherapeutic drugs while alleviating chemotherapy-related adverse effects [143,144]. Its tumour-suppressing effects occur via activation of proapoptotic factors, blockage G2/M transition, suppression of mitotic divisions, and modulation of PI3K/Akt/mTOR signalling [143,145]. Qianlie Xiaozheng decoction (QLXZD), a polyherbal formulation containing *Coix lacryma-jobi* and six other herbs, is clinically used to treat prostate cancer. The only mechanistic study on QLXZD to date reported its autophagy-related toxicity to involve inhibition of the Akt/mTOR pathway [146] in PC3 cells and PC3 xenografts mice.

*Artemisia annua* Linnaeus is another noteworthy plant that has been extensively studied as a cytotoxic agent in cancer cell lines and animal tumor models. Its efficacy for treating fever, inflammation, and malaria has also been evaluated in clinical studies. However, there are no reports to date on their clinical evaluation for cancer therapy in humans [147].

*Terminalia arjuna* is a medicinal plant indigenous to India with a long history of medicinal uses, including cancer treatment. Its potential as an adjunct for cancer therapy was reported in several in vitro and animal studies. Aqueous extract of *Terminalia arjuna* bark induced cell membrane damage and mitochondrial dysfunction in cancer cells while protecting the heart tissue against doxorubicin toxicity. Also, the experimental rats administered the leaf extracts did not show any noticeable toxicity in the measured haematological, biochemical, and histological parameters [148].

## 7. Metabolomics Approach in the Quality Control and Safety Evaluation of Herbal Exposure

Quality control and the discovery of active components are considered critical for modernizing traditional herbal medicines [149]. Pertinent quality control systems in the phytomedicine industries are necessary to distinguish any potential toxic adulterants from the raw plant materials and achieve consistency in the desired doses of active components in different batches of the product [150]. Metabolomics utilizes highly sensitive identification techniques, such as high-performance liquid chromatography (HPLC), mass spectrometry (MS) and nuclear magnetic resonance (NMR) [151,152], which are vital in the chemical fingerprinting of drugs in development. This is particularly relevant for quality control purposes of phytomedicines which are usually composed of diverse chemical compounds. Using metabolomics, the standardized metabolic fingerprint of herbal products can be generated. The peculiar metabolomics-based fingerprinting aids the identification of inconsistent composition, batch-to-batch variation in the proportion of the active components, adulteration or inclusion of undisclosed pharmaceutical ingredients that are crucial for efficacy and patient safety. The pharmacological effects of herbal medicines usually depend on both major and minor constituents. Metabolomics also aid in the cultivation of stress-resistant plants by finding novel metabolic markers of adaptability that may lead to greater yields in raw materials [153].

Plants have gained attention for their immense medicinal values and are believed to be safer due to historical usage without many well-documented toxicity concerns. However, studies have shown this may not be the case for all medicinal plants. Herb–herb or herb-drug interactions that may lead to untoward clinical consequences have been reported [154], therefore necessitating the constant need to conduct safety assessments on any medicinal plant or product irrespective of anecdotal usage. Metabolomics-based approaches are useful in investigating the metabolic fate of herbal exposures [19,155]. Knowledge of the metabolic fate of herbal medicines can help to understand the efficacy and predict their potential toxicity or adverse drug reactions (ADR) upon consumption. Metabolomics-based strategy is also useful for the discovery of the endogenous metabolites altered by herbal interventions. For example, using a metabolomics approach, a study analyzed the metabolic fates of Pu-erh tea polyphenols in urine samples of human volunteers. The altered variables were classified as intact tea polyphenols absorbed into circulation, metabolites of the absorbed polyphenols, endogenous metabolites altered due to the intake of plant-derived compounds. The altered metabolites in tea consumers were compared to the plant metabolome or the predose human metabolome [156].

The MS- and NMR-based metabolite profiling have been useful in the early detection of ADR or to determine the toxicity profiles for many traditional Chinese herbs or Chinese materia medica and pharmaceutical drugs [20,94,157,158,159]. For example, using non-targeted metabolomics, the metabolic profiles of urine samples from *Polygonum multiflorum* Thunb.-treated and untreated rats were analyzed to screen potential biomarkers of liver and kidney damage [160]. The altered metabolic markers were combined with the biochemical indices to clarify the liver and kidney injury mechanisms of the plant. In the report, LC-MS metabolomic profiles of rat plasma, urine, and faeces revealed increases in tyrosine, L-phenylalanine, phenylpyruvate, and the uremic toxin p-cresol sulfate, which is produced by the metabolism of tyrosine and phenylalanine, indicating that amino acid metabolism disorders may be involved in the liver and kidney injury caused by *Polygonum multiflorum*. In addition, metabolomics has been successfully employed to identify the idiosyncratic tissue injury induced by the different plant extracts and several metabolic biomarkers associated with organ toxicity were examined [161,162]. In the same light, this approach is used to explore the protective mechanisms of plant extracts (e.g., *Myristica fragrans* (nutmeg) extract) against toxicant-induced organ toxicity [163] and accumulation of uremic toxins in colon cancer [164].

Analysis of the metabolome offers some advantages over conventional analytical methods for evaluating toxicity. Since metabolomics is a non-invasive technique, peripheral samples such as urine, serum, or other accessible bio-fluids can be obtained before exposure to herbal extracts, during and after exposure to evaluate onset and regression of toxicity in the organism [165]. Metabolomics analysis could provide a mechanistic understanding of toxicological effects that were not apparent with traditional toxicology evaluation. Metabolomics analysis showing an increase in several measured phytosterols in an identical fashion to cholesterol increase could be indicative of sterol absorption at the gut level that was not specific to cholesterol. Also, metabolomic analysis of serum bile acid profiles can further support if the noticed changes in cholesterol levels were due to an altered bile acid kinetics [166].

## 8. Challenges

A limited number of published data exist on metabolomics applications to understand the anticancer effects of herbal medicines. Studies translating metabolomics findings into clinical applications are also limited. The clinical applications of metabolomics in investigating the anticancer efficacy of herbal therapy would benefit from an in-depth understanding of how metabolite measurements are connected to cancer biology, especially in readily accessible biofluids. Metabolite analysis to evaluate cancer cells response to herbal medicines have many challenges such as tumour heterogeneous metabolic preferences, lack of specific metabolic signature for each cancer type, the structural diversity of cancer cell and plant metabolites. Others are the challenges in distinguishing anticancer metabolic effects of the herbal exposure from general metabolic perturbations in biofluids, influence of environmental factors, genetic factors and gut microbiota, sample preparation, variation in the origin, and handling of cell lines [167,168,169]. Technological limitations specific to metabolomics may include sample preparation, standardization of instrumentation, high cost of analytical instruments, structurally diverse compounds, data processing and interpretation, availability of trained manpower, and poor publicity of metabolomics compared to other omics technologies [170,171].

Although there is more to learn on the metabolic complexity of cancer and technical aspects of metabolomic profiling, significant progress has been made in the past decade. Technical and methodological improvements are being made for metabolite analysis in tissues and biofluids to further our understanding of cancer metabolomes [172,173]. A better understanding of molecular mechanisms underlying metabolic reprogramming and tumor heterogeneity will promote a more meaningful interpretation of metabolite patterns in intervention studies. Improved understanding of microbiome–metabolome interaction in human malignancies—an area currently under intense investigation—may also further our understanding of cancer metabolic adaptability and the development of targeted therapies [174]. The majority of cancer metabolomics studies of medicinal plants to date were conducted using cell lines. While cell lines are invaluable to investigate metabolic regulatory mechanisms of herbal medicines, systems that can recapitulate the genetic heterogeneity and microenvironment of human tumors are also needed. Significant progress has been made to develop metabolome databases and improve metabolite coverage for metabolite identification and data visualization [175,176]. Identification of novel cancer type-specific metabolomic signatures and their clinical relevance will promote the application of metabolomics studies for cancer drug development from traditional herbal medicines.

## 9. Conclusions

Cancer metabolism is influenced by a complex set of factors; hence, a comprehensive metabolic analysis is indispensable to unravel the anticancer actions of metabolic-targeting plants. Metabolomics has an advantage from a translation standpoint as it directly conveys phenotype and has a wide application from single-cell experiments to complex clinical studies. The holistic approach of metabolomics fits traditional herbal medicine’s holistic concept and offers an opportunity to gain a comprehensive mechanistic insight into the anticancer efficacy of traditional herbal medicine. Although there are still many hurdles to overcome, metabolomics offers a promising comprehensive approach to identify the effects of herbal interventions on metabolite pattern, enzyme activity, and possible alterations at the DNA, RNA, and protein levels. Integration of data obtained from metabolomics studies with those from other omics techniques—genomics, transcriptomics, proteomics—could provide a functional relationship between metabolite changes and the molecular drivers of malignant transformation. Metabolomics would also facilitate the standardization of traditional herbal medicines.

## Figures and Tables

**Table 2:** Pharmacological agents targeting cancer metabolic vulnerabilities.

Metabolic Target	Drug	Study Phase	Mode of Action	Reference
Glycolysis inhibitors	2-deoxy-D-glucose	Phase III clinical trial	Competitively inhibits glucose uptake by interfering with HK	[54]
	WP1122—Novel 2-DG analog)	Phase II clinical trial	Inhibits HK	[55]
	AZD3965	Phase II clinical trial	Inhibits MCT 1 causing lactic acid accumulation and feedback inhibition of glycolysis	[56]
Pyruvate dehydrogenase complex inhibitor	Dichloroacetate	Phase II clinical trial	Inhibits PDHK and reactivate the TCA cycle	[57]
Isocitrate dehydrogenase inhibitor	Enasidenib—novel IDH inhibitor	Phase III clinical trial	Inhibits mutant IDH2 variants and lowers serum levels of 2-HG in acute Myeloid Leukemia	[58]
Glutamine transport Inhibitor	V-9302	Preclinical data	Selectively inhibits ASCT2 transporter	[59]
Fatty acid synthesis inhibitor	TVB-2640	Phase 1 clinical trial	Inhibits FASN	[60]
	Cerulenin	Preclinical data	Inhibits FASN	[61]
	Fatostatin	Preclinical data	inhibits SREBP activation	[62]
	A939572	Preclinical data	Inhibits SCD-1	[63]
Mdm2 inhibitors	Idasanutlin (RG73388)	Phase III clinical trial	Inhibits MDM2-p53 interaction	[64]
	AMG-232	Phase I clinical trial	Blocks MDM2-p53 interaction	[65]

Abbreviations: Hexokinase (HK), Monocarboxylate transporter (MCT), Pyruvate dehydrogenase kinase (PDHK) Isocitrate dehydrogenase (IDH), Alanine-serine-cysteine transporter (ASCT), stearoyl-CoA desaturase (SCD).

**Table 3:** Modulatory effects of medicinal plants on cancer metabolism.

Target Classification	Metabolic Process	Target	Metabolic Effects of Plants and Derived Compounds on Cancer Cells and Tumour
Enzymes	Glycolysis	Hexokinase 2 (HK2)	*Annona muricata* Linnaeus (Graviola) ethanolic extract reduced HK2 protein expression and other proteins related to glycolysis in pancreatic cancer [67].
		Phosphofructokinase (PFK)-1 & 2	Oleanolic acid found in the Oleaceae plant family reduced aerobic glycolysis and proliferation in human MKN-45 and SGC-7901 gastric cancer cells via reduced expression and intracellular activities of PFK-1 and HK2 [68].
		Pyruvate kinase (PKM2)	*Carpesium abrotanoides* Linnaeus. root extract reduced glycolysis by downregulating the expression of PKM2 and inhibited PKM2/HIF-1α in breast cancer cells [69].
	Pyruvate metabolism Pyruvate metabolism	Pyruvate dehydrogenase (PDH)	Resveratrol markedly increased PDH complex activity in colon cancer (Caco2) cells [70].
		Pyruvate dehydrogenase kinase (PDHK)	*Cinnamomum cassia* (Linnaeus) J. Presl aqueous extracts induced apoptosis by inhibiting PDHK activity and promoting a metabolic shift from glycolysis to oxidative phosphorylation by reducing protein expression of phosphorylated PDH in human lung cancer cells (A549 cells and H1299) and murine Lewis lung carcinoma cells (LLC) [71]. *Anemone rivularis* Buch. Ham. ex DC. whole plant ethanol extract inhibited aerobic glycolysis by reducing phosphorylation of PDHK in human colon cancer (DLD-1) and murine cells (LLC) [72]. Huzhangoside A- a triterpenoid glycoside from several plants of the genus-*Anemone,* inhibited PDHK1 activity, and induced the apoptosis of colorectal adenocarcinoma cell lines [73].
		Pyruvate dehydrogenase phosphatases (PDP)-1 & 2	Resveratrol exposure significantly enhanced the expression of PDP-1 mRNA in colon cancer (Caco2) cells. [70].
		Pyruvate carboxylase (PC)	1,2,3,4,6-penta-O-galloyl-beta-d-glucose (PGG)- a compound from *Rhus chinensis* Mill. downregulated *PC* gene in MDA-MB-231 human breast cancer cells [74].
	Lactate metabolism	Lactate dehydrogenase A (LDHA)	*Annona muricata* (Graviola) extract reduces LDHA protein expression in pancreatic cancer cells and significantly suppressed cell proliferation [67]. *Spatholobus suberectus* Dunn aqueous extract inhibits LDHA activity in human breast cancer cells [75]. *Myristica fragrans* Houtt. seed aqueous extracts suppressed the growth of human colon cancer cell (HT29) by reducing the activity of LDHA and lactate production [76].
	Fatty acid synthesis	ATP citrate lyase (ACLY)	Cucurbitacin B, a compound from cucumber, inhibited the phosphorylation of ACLY and suppressed the growth of prostate cancer cells (PC-3 and LNCaP) and tumor-formation in a chemopreventive prostate tumor mouse model [77].
		Acetyl-CoA carboxylase (ACC)	Andrographolide, a labdane diterpenoid extracted from the rhizomes of *Andrographis paniculata* (Burm.f.) Nees, suppressed MV4-11 cell proliferation and blocked fatty acid synthesis by downregulating FASN and ACC expression [78].
		Fatty acid synthase (FASN)	Extra-virgin olive oil-derived phenolics (lignans, secoiridoids, and flavonoids) suppressed the expression of FASN protein in HER2 over-expressing breast cancer cells [79].
Transporters	Glucose transport	GLUT1/4	*Annona muricata* extract reduced GLUT1/4 protein expression levels in metastatic PC cell lines FG/COLO357 and CD18/HPAF [67].
	Monocarboxylate transport	Monocarboxylate transporter (MCT) 1–4	*Terminalia chebula* Retz. fruit extract reduced the expression of MCT1, MCT3, MCT4, and their chaperone CD147 in mouse brain neuroblastoma cells (N2-A) and induced apoptotic cell death [80].
	Amino acid transport	Alanine-serine-cysteine transporter (ASCT) 2	Ursolic acid in combination with either curcumin or resveratrol reduced protein expression of ASCT2 in HMVP2 prostate cancer cells [81].
Oncogenes and Tumor suppressors	Cell signalling and growth regulation	*MAF*	Ricinus extract downregulated MAF oncogene in MCF7 human breast cancer cells [82].
		C-MYC	*Azadirachta indica* A.Juss. ethanolic leaf extract suppressed c-Myc oncogene expression in 4T1 breast cancer BALB/c mice [83].
		PI3K/AKT	Betulinic acid, a pentacyclic lupane triterpene, promoted apoptosis by downregulating PI3K/AKT signaling in HeLa cells [84]. Emodin from *Rheum officinale* Baill. and *Polygonum cuspidatum* Sielbold & Zucc. inhibited PI3K/AKT and ERK signaling in triggered apoptosis in human hepatocellular liver carcinoma cells (HepG2) cells [85].
		MTORC1	*Remotiflori radix* ethanol extract suppressed the growth of PC-3 cells by increasing phosphorylated AMPK expression and inhibiting mTOR activation [86]. Curcumin inhibited the mTOR-HIF1α axis [87].
		p53	*Tulbaghia violacea* Harv. extracts increased p53 protein expression and induced apoptosis in MRC-5 HeLa, MDA-MBA-231, MCF-7 and ME-180 cell lines [88]. Platycodin D from *Platycodon grandiflorus* (Jacq.) A.DC. decreased the protein levels of MDM2 and mutant p53 in MDA-MB-231 cells and xenograft model [89].
		Phosphatase and tensin homolog (PTEN)	Thymoquinone isolated from *Nigella sativa* Linnaeus (L) oil increased PTEN mRNA and protein expression in doxorubicin-resistant MCF-7 cells and induced G2/M phase arrest and apoptosis through upregulation of p53 expression and decreased AKT phosphorylation [90]. Resveratrol increased the protein expression of PTEN and phosphorylated p53 in NALM-6 acute lymphoblastic leukemia cells, resulting in a decrease in the activation of AKT and ERK [91].
Transcription factors	Gene transcription	Sterol regulatory element-binding protein (SREBP)	Betulinic acid decreased SREBP-1 activity, activates CaMKK, and up-regulates AMPK activity by phosphorylation, which results in reduced lipogenesis and lipid accumulation in HepG2 cells [92].
		Hypoxia-inducible factor (HIF)- 1	Celastrol from *Tripterygium wilfordii* Hook. f. root extracts reduced hypoxia-induced angiogenic and metastatic activity in human umbilical vein vascular endothelial cells (EA.hy926) and HepG2 cells by decreasing HIF-1α mRNA and protein levels and transcriptional activity of HIF-1α target genes [93].
		Signal transducers and activators of transcription (STAT) 3	Pectolinarigenin from *Cirsium chanroenicum* (Nakai) Nakai impaired cancer cell migration and invasion by down-regulating the expression of p-STAT3, MMP-2, and MMP-9 in human breast cancer cells MDA-MB-231, MCF-7 [94].

## Data Availability

Not applicable.
